# Unexpected Adverse Events of Immune Checkpoint Inhibitors

**DOI:** 10.3390/life13081657

**Published:** 2023-07-29

**Authors:** Walid Shalata, Alexander Yakobson, Aharon Y. Cohen, Iris Goldstein, Omar Abu Saleh, Yulia Dudnik, Keren Rouvinov

**Affiliations:** 1 The Legacy Heritage Oncology Center & Dr. Larry Norton Institute, Soroka Medical Center, Ben-Gurion University, Beer Sheva 84105, Israel; 2 Department of Neurology, Faculty of Health Sciences, Soroka Medical Center, Ben-Gurion University, Beer Sheva 84105, Israel; 3 Department of Dermatology and Venereology, The Emek Medical Centre, Afula 18341, Israel

**Keywords:** pembrolizumab, palmoplantar keratoderma, Reiter’s syndrome, myasthenia gravis, autonomic neuropathy, immune related adverse events, immune checkpoint inhibitors

## Abstract

The introduction of immune checkpoint inhibitors (ICIs) has revolutionized cancer treatment standards and significantly enhanced patient prognoses. However, the utilization of these groundbreaking therapies has led to the observation and reporting of various types of adverse events, commonly known as immune-related adverse events (irAEs). In the following article, we present four patients who encountered uncommon toxicities induced by ICIs. The first patient was a 59-year-old female diagnosed with stage 4 lung adenocarcinoma. She received immunotherapy (pembrolizumab) together with chemotherapy and subsequently developed autonomic neuropathy (AN). The next two patients also received chemo-immunotherapy (pembrolizumab) and were both 63-year-old males with stage 4 lung adenocarcinoma. One of the two experienced palmoplantar keratoderma, while the other presented with Reiter’s syndrome (urethritis, conjunctivitis and arthritis). The 4th patient, an 80-year-old male with stage 4 squamous cell carcinoma of the lung, received chemo-immunotherapy (pembrolizumab) and developed myasthenia gravis.

## 1. Introduction

Immune checkpoint inhibitors (ICIs), or immunotherapy, has revolutionized the standard of care of various malignancies, establishing new standards of care in neo-adjuvant, adjuvant and metastatic treatment settings. One notable ICI is pembrolizumab, which has gained approval for the management of multiple malignancies such as colon cancer, melanoma, non-small cell lung cancer (NSCLC) and renal cell cancer. Nonetheless, the utilization of ICIs has been associated with a diverse range of adverse events, known as immune related adverse events (irAEs) [1]. ICIs are antibodies that target suppressive proteins, including CTLA-4 and PD-1/PD-L1, found on immune cells [2]. Pembrolizumab (keytruda) is a humanized Ig-G4 monoclonal antibody that targets a key immune-checkpoint receptor involved in regulating T-cell response. It, along with nivolumab, another ICI, functions by inhibiting the programmed cell death receptor 1 (PD-1). By inhibiting the activities of PD-1, these ICIs enhance the anti-tumor T-cell response; however, the enhanced proliferation of T-cells can also give rise to irAEs [3,4]. The importance of ICIs lies in their potential to achieve long-term, and even complete, responses in some patients. However, the reality is that the majority of patients still do not benefit from these treatments. Determining whether a patient is experiencing a genuine clinical impact from immune-oncology (IO) drugs can be challenging for physicians, especially when concepts like pseudoprogression or hyperprogression come into play. These concepts differ significantly from the classical response patterns observed with conventional oncology therapies. One crucial aspect to consider is that ICI toxicity does not seem to be associated with the drug dose, and thus, dose reductions are unlikely to prevent them from occurring. As a result, immune-related toxicities frequently necessitate permanent discontinuation of treatment, especially when ICI combination strategies are employed [5].

An analysis of almost 3953 patients across multiple clinical trials showed that the most common irAEs were diarrhea, rash, myalgia, nausea, pneumonitis and arthralgia. Apart from rash, additional cutaneous complications included pruritus and, less frequently, vitiligo [6]. Increasingly, ICIs have been recognized as being associated with neurological adverse events, often presenting with sub-acute and variable symptoms and signs [7]. Although neurologic irAEs may occur less frequently than other irAEs, there is still a potential for substantial long-term morbidity and, in some cases, even mortality [8]. Autonomic neuropathy (AN) is an extremely rare complication of ICI treatment. Severe immune-related toxicities of the central nervous system account for 0.4% of the overall incidence of irAEs [9]. Palmoplantar keratoderma (PPK) is characterized by persistent thickening of the skin of the soles and palms. PPK can either occur as an acquired condition or be associated with genetic factors. In some cases, PPK may manifest as a paraneoplastic syndrome, in which a dermatosis appears in a distant location in relation to an internal malignancy [10]. Paraneoplastic PPK has been observed in association with various visceral malignancies, including gastric, esophageal, bladder (urinary) and pulmonary carcinomas [11]. Reiter’s syndrome, a reactive arthritis, is a classic triad of urethritis, arthritis and conjunctivitis. It is typically associated with infections of the urogenital tract or gastrointestinal and may also involve the sacro-iliac joints [12].

To our knowledge, there have been no previous reports of Reiter’s syndrome and palmoplantar keratoderma being associated with anti PD-1 treatment for lung cancer. Autonomic neuropathy and myasthenia gravis are rare secondary effects of ICIs treatment for lung cancer. In this report we present the cases of four patients, who developed autonomic neuropathy, palmoplantar ketatoderma, Reiter’s syndrome and mysasthenia gravis, respectively. Each case adds valuable insights to our understanding of the diverse and evolving spectrum of irAEs associated with immunotherapy and the management strategies that can be used for it. This information can aid in improving patient care and guiding future research to optimize treatment strategies and manage potential complications effectively.

## 2. Rare and Exclusive Cases Description

### 2.1. Severe Autonomic Neuropathy Induced by Pembrolizumab

A 59-year-old female was admitted to Soroka Medical Center in the beginning of January 2019 due to the suspicion of pneumonia, and received antibiotic treatment. She had an almost 30 pack-year history of smoking, but had stopped smoking 20 years previously. Her past medical history included stage 1 malignant melanoma that was surgically removed approximately 30 years ago in the area of right upper extremity and papillary thyroid cancer for which she underwent thyroidectomy almost 7 years ago. Chest radiography (CXR) almost one month later revealed the same ground-glass opacity (GGO) in the right upper lung (RUL). A chest computed tomography (CT) scan revealed a 2.9 cm ground-glass opacity in the RUL, enlargement of the right hilar lymph nodes to 1.3 cm and a 1.3 cm space-occupying lesion (SOL) in the right middle lung (RML).

She was referred to the oncology center for further evaluation. Two biopsies were obtained (RML mass and from the RUL opacity) through mediastinoscopy, and the histopathological results showed adenocarcinoma (ADC) of lung origin (NSCLC). Positron emission tomography (PET-CT) scan revealed increased hypermetabolic absorption in the RUL GGO and RML SOL, diffuse small lesions up to 0.5 cm that were suspected to be metastases, and right hilar lymph nodes, establishing the diagnosis of stage 4 (T2N2M1) adenocarcinoma of the lung. MRI of the head ruled out any signs of brain metastasis. Molecular testing showed the programmed death ligand-1 (PDL-1) value at 50%, and was negative for mutations of EGFR, BRAF, ROS1, MET, RET and ALK rearrangement. Consequently, the patient was started on the intravenous chemotherapy–immunotherapy regimen of pemetrexed (500 mg per m^2^) and carboplatin (area under the curve (AUC) of 5), along with pembrolizumab (fixed dose of 200 mg) on day 1 every 21 days.

**Figure 1:**
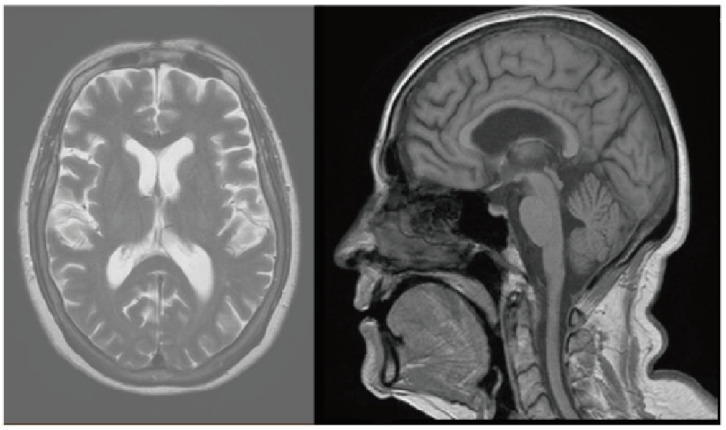
MRI of the head revealed no pathologic findings or evidence of metastasis.

Two weeks after the administration of the third cycles of treatment, the patient presented with sweating, hot flashes, vertigo, headaches, pain in both arms and systemic chills. It was decided to discontinue the treatment, and she was hospitalized for further investigation. MRI of the spine and head was performed, which showed no evidence of distant metastasis or other abnormalities (Figure 1). Bacterial growth was ruled out by negative results of urine and cultures. CT scan of the total body did not indicate the source or the cause of the fever. Echocardiography revealed no signs of myocarditis or pericarditis, with normal range of ejection fraction. Due to bilateral upper extremity paresthesias and arm pain, the patient was referred for neurological consultation, and a diagnosis of suspected autonomic neuropathy from pembrolizumab therapy was made.

Treatment with steroids, prednisone (1 mg per kg), was initiated and continued for 2 weeks. Since there was no observed improvement in the symptoms despite the treatment regimen, it was decided to increase the prednisone dose to 1.5 mg/kg, and treatment was continued for an additional two weeks. Despite steroid treatment, the symptoms persisted, and the patient was readmitted to our department and received intravenous immunoglobulin (IV-IG) treatment at a daily dose of 0.4 mg per kg for five days. Several days after starting IV-IG therapy, she reported relief from the paresthesias and arm pain. Upon completing the fifth day of IV-IG treatment, the symptoms had almost completely resolved, and the episodes of hot flashes and sweating had completely ceased.

### 2.2. Palmoplantar Keratoderma Induced by Pembrolizumab

A 63-year-old male with a history of 40 pack-years of smoking and hypertension (HTN) was admitted to the hospital at the end of June 2017 due to worsening cough and discomfort in the middle chest region. A chest CT scan revealed significant enlargement of lymph nodes, measuring 2.3 cm in the hilum of the left lung, 1.3 cm in hilum of the right lung and 3 cm in the mediastinal area. Additionally, a GGO with a diameter of 2.2 cm was observed in the left upper lung (LUL).

For further investigations, he was referred to the oncology center. A PET-CT scan showed hyper-metabolic absorption of the lymph-nodes (LN) and the previously observed GGO. Hyper-metabolic absorption was also noted in the retroperitoneal LN, close to the portal vein, measuring almost 1.9 cm in diameter, as well as in a 1.5 cm area of the RT iliac bone. MRI of the head revealed no pathologic findings. A biopsy from the mass in the LUL was performed under CT guidance, confirming the diagnosis of ADC originating from the lung. The stage was determined as stage 4 (T2,N3,M1) lung ADC. Molecular testing showed the PDL-1 value at 35% and was negative for ALK-rearrangement and EGFR-mutation. Based on those results, he received systemic chemotherapy–immunotherapy with cisplatin (75 mg per m^2^), and pemetrexed (500 mg per m^2^), plus pembrolizumab (fix dose of 200 mg) on the first day every 21 days. After the third cycle, there was a radiological partial response (PR). It was then decided, due to the severe nausea, to switch the carboplatin instead of the cisplatin. After completing six cycles of platinum-containing therapy, the patient remained on treatment with pemetrexed and pembrolizumab for an additional five cycles. The systemic therapy had to be discontinued due to the development of interstitial pneumonia, which was suspected to be caused by pembrolizumab.

Later (after 2 months), the pembrolizumab was renewed at the same dose and frequency due to a PET-CT scan which revealed increased metabolic absorption in the region of the right supraclavicular LN and lymphadenopathy in the hilum of the left lung. 

**Figure 2:**
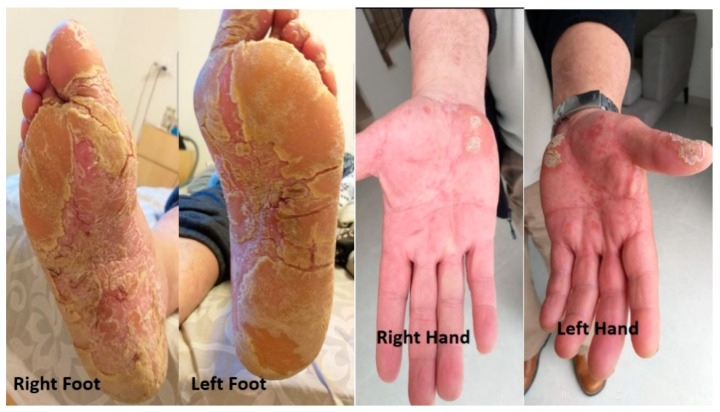
Palmoplantar keratoderma of hands and feet observed prior to discontinuing pembrolizumab and initiating dermatological treatment.

A year after resuming the treatment, the patient experienced cutaneous changes that were characterized by thickening and peeling of the skin in both hands and feet. He also experienced moderate and progressive pain in conjunction with the cutaneous changes. A dermatologist suspected palmoplantar keratoderma (PPK) (Figure 2).

**Figure 3:**
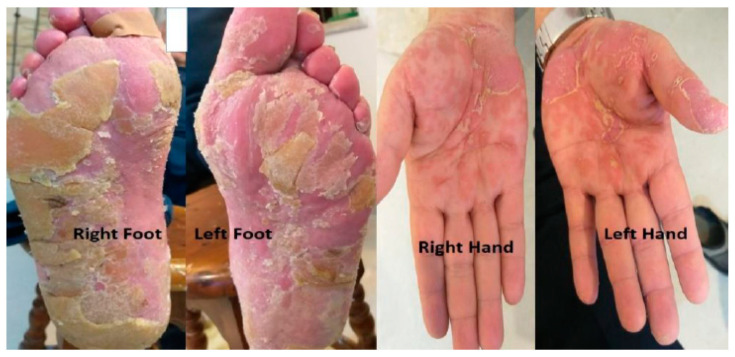
Palmoplantar keratoderma improvement after stopping pembrolizumab and using the dermatological treatment.

In an attempt to mitigate this adverse event, we implemented an interval adjustment between pembrolizumab administrations, extending it to every six weeks while keeping the same dose. However, despite this effort, two months later, pembrolizumab had to be discontinued due to the exacerbation of pain, despite improvement in the physical findings of PPK. (Figure 3). 

**Figure 4:**
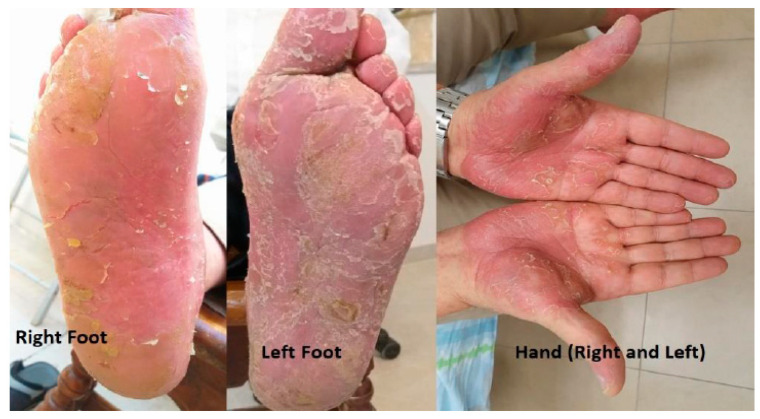
During dermatological therapy, a notable improvement in palmoplantar keratoderma on the hands and feet was observed.

The patient was again seen in consultation by the dermatologist for further evaluation and management, and topical salicylic acid 10% was initiated, along with oral acitretin (Aci-tretin™) at a daily dose of 25 mg and bifonazole urea cream (Keratospor™). Two months later, there was regression of skin thickening in the extremities, along with significant improvement of the pain. (Figure 4).

### 2.3. Reiter’s Syndrome Induced by Pembrolizumab

A 63-year-old male was in routine follow-up for localized laryngeal squamous cell carcinoma (stage T2-N0-M0) diagnosed in 2001. The patient was a heavy smoker with a history of hypothyroidism and a cerebrovascular accident (CVA). In September 2018, a CT scan of the head was performed due to recurrent falls and headache, revealing a single right occipital brain metastasis measuring 2.2 cm in diameter accompanied by surrounding edema. Total body CT scan showed a mass in the RUL of the lung measuring 1.6 × 1.5 cm and LN enlargement up to 1.5 cm in the right hilar region. An RT occipital craniotomy was performed to remove the brain metastasis, and a segmental resection of the right upper lobe (RUL) was conducted. Histopathologic examination of both areas confirmed that the tumor originated from the lung, and it was classified as ADC stage 4 (T1-N1-M1). Following that surgery, the patient received radiotherapy to the brain (craniotomy area), with a total dose of 30 Gy delivered in 10 fractions. Molecular testing showed a highly positive PDL-1 value at 50%, and was negative for ALK-rearrangement and EGFR-mutation. Systemic treatment was initiated with immunotherapy and chemotherapy, which consisted of carboplatin (of AUC -4) and vinorelbine (30 mg per m^2^) administered on the first and eighth days, plus pembrolizumab (200 mg) on day 1 every 21 days for four cycles. The patient showed a complete radiological response, with the mediastinal LN regressing to normal size, and no signs of metastatic disease were noted.

After completing those initial cycles of chemo-immunotherapy, the patient continued pembrolizumab, only as maintenance therapy, which was given at the same dosage and frequency. However, following the eighth cycle, the patient developed several symptoms: eye irritation, burning sensation while urinating and knee pain. On physical examination, conjunctivitis in both eyes was noted, along with swelling and tenderness in both knees. Blood tests (chemistry and complete blood count) showed no abnormalities. 

Based on the clinical presentation and examination findings, a multidisciplinary conference was conducted involving specialists from various fields, including oncology, rheumatology, immunology and infectious diseases. The conference concluded that the clinical presentation was consistent with grade three conjunctivitis and grade two arthritis, as well as suspicious for Reiter’s syndrome. These symptoms were determined to be most likely attributable to the immunotherapy treatment with pembrolizumab. As a result, pembrolizumab was discontinued. 

The patient underwent further investigations, including genitourinary cultures neisseria gonorrhoeae, ureaplasma urealyticum, chlamydia trachomatis and mycoplasma hominis, as well as genitourinary serological tests, all of which were negative. There was no clinical evidence of gastrointestinal disease, and there were no symptoms or signs of infection. 

To alleviate the symptoms, corticosteroid therapy was initiated using prednisone at a dosage of 1.5 mg/kg. The patient underwent a two-week treatment course, which led to notable improvement in the symptoms’ disappearance.

### 2.4. Severe Myasthenia Gravis Induced by Pembrolizumab

In March 2020, an 80-year-old male was admitted to the hospital with a persistent cough and dyspnea of almost four weeks’ duration. Additionally, the patient had been experiencing left-sided chest pain over the previous two months. He had a history of heavy smoking (40 pack-years), but had stopped eight years previously. He had also been diagnosed in the past with type 2 diabetes and HTN. CXR showed GGO in the LUL of the lungs, a diagnosis of pneumonia was made and the patient received antibiotics. After four weeks, follow-up CXR showed persistence of the GGO in the LUL. Consequently, an endo-bronchial ultrasound was performed, followed by a biopsy of the suspicious findings in the LUL. The pathological examination confirmed the presence of squamous cell carcinoma originating from the lung. Further investigations were conducted to determine the extent of the disease. MRI of head revealed no evidence of malignancy, while a PET-CT scan showed hyper-metabolic activity in the LUL solid lesion (measuring 6.5 × 8.5 cm) and left mediastinal lymphadenopathy (up to 2 cm). Additionally, multiple lung nodules were identified, measuring up to 4–6 mm in diameter, which were too small to exhibit uptake on the PET scan, but which were suspected to be lung metastases. 

Based on the clinical assessment, the patient was diagnosed with stage 4 (T4-N1-M1) NSCLC. Molecular testing showed highly positive PDL-1 (50%), and was negative for ALK-rearrangement and EGFR-mutation. A systemic intravenous chemotherapy–immunotherapy regimen was initiated consisting of carboplatin (AUC of 3) and paclitaxel (at a dosage of 175 mg per m^2^), plus pembrolizumab administered at a fixed dose of 200 mg on the 1st day every 21 days. Following the completion of four cycles of treatment, a PET-CT scan was repeated to evaluate the response. The results demonstrated a positive radiological response, as evidenced by a reduction in the size of the LUL mass to 6 × 4 cm. Moreover, there was a decrease in the hypermetabolic activity observed in the lymphadenopathy of the mediastinum, along with regression of the LNs to normal diameter. Additionally, the lung nodules were no longer detectable.

**Figure 5:**
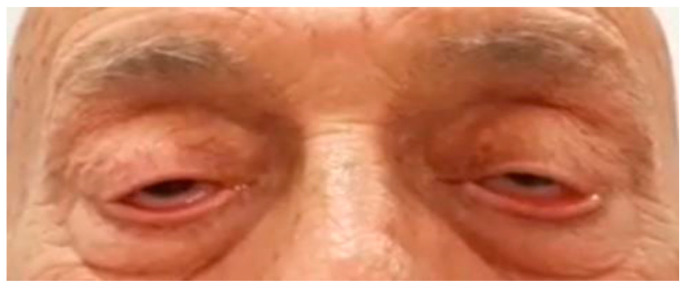
Patient at presentation, showing bilateral ptosis (drooping of the eyelids) along with near-complete ophthalmoplegia (paralysis or weakness of eye movements).

The patient continued to receive pembrolizumab, as a maintenance monotherapy, at the same dose and frequency. Following the second cycle of the treatment, the patient reported symptoms of bilateral ptosis (drooping eyelids) with near complete ophthalmoplegia (paralysis or weakness of eye muscles), (Figure 5), dysarthria, and asymmetry of the left corner of the mouth. A CT of the brain was performed which ruled out malignancy and CVA.

The suspected diagnosis was myasthenia gravis as an IrAE induced by pembrolizumab. Therefore, the decision was made to discontinue pembrolizumab. The patient underwent a neurology consultation, which confirmed the diagnosis of myasthenia gravis.

**Figure 6:**
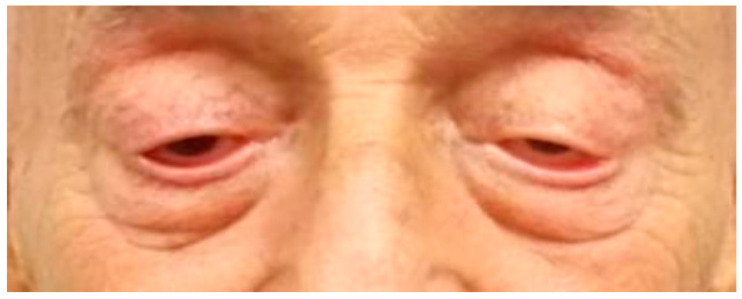
Depicts the patient after undergoing corticosteroid therapy with prednisone at a dose of 1 mg/kg for a period of two weeks.

For treatment of the myasthenia gravis symptoms, corticosteroid therapy was initiated with prednisone at a dose of 1 mg/kg for a period of two weeks. The patient experienced only minimal clinical improvement by the conclusion of the treatment period (Figure 6).

**Figure 7:**
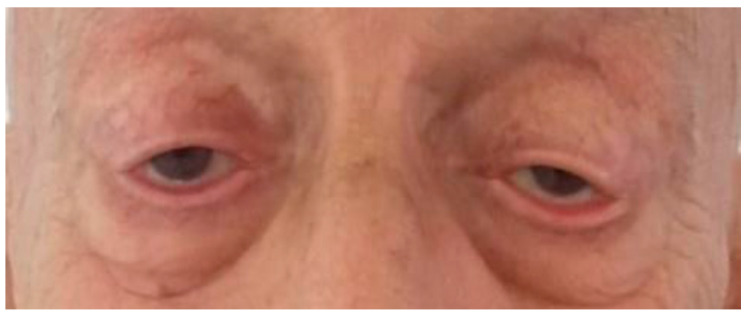
Depicts the patient’s condition, and an improvement of bilateral ptosis following the 2nd day of IV.IG.

**Figure 8:**
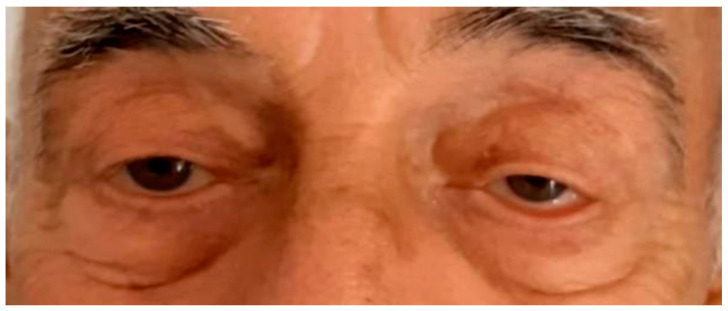
Depicts the patient’s condition, and the improvement of bilateral ptosis immediately after IV-IG therapy.

Subsequently, the patient was readmitted to the department for an alternative treatment approach. He was given IV-IG therapy at a daily dose of 0.4 mg per kg for a duration of 5 days. IV-IG involves the administration of high-dose immunoglobulins intravenously. After the second day of therapy, an improvement was noted in ptosis, and his speech became clearer (Figure 7 and Figure 8). Over the course of the following week, the symptoms completely resolved.

## 3. ICI Therapies

### 3.1. The Anti-PD-1 ICI’s

#### 3.1.1. Pembrolizumab

Pembrolizumab is a humanized IgG4 monoclonal antibody that specifically targets PD-1. Unlike regular IgG antibodies, this particular antibody, pembrolizumab, does not activate antibody-dependent cellular cytotoxicity.

Pembrolizumab has received United States Food and Drug Administration (FDA) approval for treating several types of cancer, such as urothelial carcinomas (UC), malignant melanoma (MM), renal cell carcinoma (RCC), NSCLC, Merkel cell carcinoma (MCC) and different types of squamous cell cancers (SCC). In the approval for pembrolizumab, it was noted that irAEs occur in approximately 70% of treated patients.

The irAEs due to pembrolizumab include hypo- or hyperthyroidism, constipation, diarrhea, fever, cough, fatigue, rash, pruritus and, in some cases, musculoskeletal pain (Table 1) [2,4,13,14]. 

#### 3.1.2. Nivolumab

Nivolumab is another humanized IgG4 monoclonal antibody that specifically targets PD-1. Nivolumab is similar to pembrolizumab in that it does not work through the pathway of antibody-dependent cellular cytotoxicity. It has received FDA approval for treating several types of cancer, such as NSCLC, colorectal cancer, TCC, MM, mesothelioma, lymphoma, RCC and hepatocellular carcinoma (HCC) and esophageal SCC. IrAEs associated with nivolumab include vomiting, nausea, hypo- or hyperthyroidism, constipation with abdominal pain, diarrhea, rash, pruritus and, in some cases, dyspnea or cough. Though rare, fever and headache have been reported. 

It is known that the development of irAEs associated with nivolumab can occur even after a prolonged period after completing therapy. This delayed onset may be attributed to the long-lasting effects of a single administration of nivolumab, which can result in the inactivation of PD-1 molecules for approximately three months. It has been observed that the combination of nivolumab plus ipilimumab has a higher incidence of AEs compared to that of either agent used alone (Table 1) [2,13,15,16].

#### 3.1.3. Cemiplimab

Cemiplimab is another humanized IgG4 monoclonal antibody that specifically targets PD-1. Its mechanism of action is similar to those of nivolumab and pembrolizumab. It has received FDA approval for treating several types of cancer, such as basal cell carcinoma, NSCLC and cutaneous SCC. In patients receiving cemiplimab, the most common irAEs include nephritis, pneumonitis, hepatitis, rash, hypo- or hyperthyroidism, pruritus and colitis (Table 1) [2,13,17]. 

### 3.2. The anti-PDL-1 ICI’s

#### 3.2.1. Atezolizumab

Atezolizumab is a monoclonal humanized IgG antibody that specifically targets PD-L1.

It has received FDA approval for treating several types of cancer, such as NSCLC, TCC, MM, breast cancer (triple negative type) and HCC.

The most common irAEs of atezolizumab are dyspnea, hypo- or hyperthyroidism, nausea, fatigue, headache, rash, alopecia, decreased appetite, vomiting, diarrhea, constipation and cough. Although rare, atezolizumab can cause severe and potentially life-threatening events, including toxic epidermal necrolysis, acute generalized exanthematous pustulosis, drug rash with eosinophilia and systemic symptoms, and also Stevens–Johnson syndrome (Table 1) [2,13,18].

#### 3.2.2. Durvalumab

Durvalumab, similar to atezolizumab, is a monoclonal humanized IgG antibody that specifically targets PD-L1. It has received FDA approval for treating several types of cancer, such as TCC, NSLC and HCC. The most-commonly reported irAEs of durvalumab are dyspnea, pneumonitis, cough, endocrinopathies including hypo- or hyperthyroidism, constipation and diarrhea (Table 1) [2,13,19]. 

### 3.3. The anti-CTLA-4 ICI

Ipilimumab

Ipilimumab is a fully human recombinant monoclonal antibody that specifically targets CTLA-4 (cytotoxic T-lymphocyte-associated protein 4). It has received FDA approval for treating several types of cancer, such as NSCLC, colorectal cancer, MM, RCC and HCC. Ipilimumab is rarely administered as a single agent (monotherapy), and is typically used in combination with nivolumab. This combination has shown increased response rates compared to ipilimumab as monotherapy; moreover, the ipilumimab–nivolumab combination has a greater incidence of AEs compared to either ipilumimab or nivolumab administered alone. Due to its mechanism of action in immune system stimulation, ipilimumab does not typically cause common cytotoxic chemotherapy side effects like bone marrow suppression. Up to 90% of patients may experience irAEs, including rash, endocrinopathy, colitis, pruritus, dermatitis, diarrhea, hepatitis, fatigue and neuropathy (Table 1) [2,13,20] 

## 4. Discussion

It appears that the spectrum of neurologic complications from either immunotherapy or the ICI has been widely expanding, and presently includes a painful brachial plexus neuritis, which refers to the inflammation of the network of nerves that control the movement and sensation of the upper limbs [21,22,23,24,25]. Reiter’s syndrome, an autoimmune disorder, is characterized by a triad of symptoms, which include conjunctivitis, genitourinary symptoms and arthritis. The most common underlying causes associated with Reiter’s syndrome are intestinal or genital infections [26].

In the presented cases, it was hypothesized that the symptoms experienced by the patients were related to an autoimmune response triggered by ICI (pembrolizumab). The treatment provided resulted in either complete symptom relief or significant improvement in these unusual cases of immune-related adverse events (irAEs).

The prognostic significance of irAEs in relation to the incongruity between progression-free survival and overall survival may vary due to several factors. It is important to note that irAEs often require treatment, and suboptimal management of these AE could potentially compromise the treatments’ overall benefit. While it is speculated that the immune activation associated with most irAEs may be linked to the immune response against tumors, evidence suggests that certain irAEs may have mechanisms unrelated to anti-tumor activity. These mechanisms may involve factors such as the microbiome, viral infections, or tissue-specific factors [4,25].

The wide range of irAEs affecting various organ systems makes recognizing treatment-related adverse events challenging, particularly in rural areas with long distances between patients’ primary healthcare units and tertiary oncology centers. There are multiple confounding factors that can complicate retrospective prognostic assessments, with one major factor being a time-related bias. Patients with a more favorable overall prognosis tend to have more time to experience different side effects of the treatment [26].

Cutaneous irAEs are prevalent in ICI therapy, accounting for over 50% of all reported irAEs. However, they are typically mild and rarely lead to severe complications that would hinder the continuation of treatment. Non-specific maculopapular rashes often emerge within the first six weeks of therapy and may be accompanied by itching (pruritus). In some cases, pruritus alone can indicate a skin AE such as bullous pemphigoid. These maculopapular rashes usually affect less than 30% of the body’s surface area and are considered severe (grade > 3) in less than 5% of cases. Under treatment with PD-1 agents, lichenoid eruptions in the forms of erythematous papules or plaques occur more frequently (≤30%) compared to anti-CTLA-4 therapy. Among ICI-related skin toxicities, maculopapular rash with or without pruritus is the most commonly observed presentation. These skin toxicities typically manifest in the weeks following the initiation of ICI therapy [8,14,27,28]. For cases of ICI-related maculopapular rash graded as 1, the ICI treatment can usually continue, and the toxicity can be managed with topical steroids, oral antihistamines, topical emollients, and appropriate investigations to rule out other causes. In grade 2 cases, temporary interruption of ICI therapy should be considered, along with the use of higher-potency topical steroids, supportive medications and systemic steroids, if needed for refractory cases. Grade 3 cases involve a rash covering more than 30% of the body’s surface area, with moderate symptoms. In addition to topical and supportive medications, oral prednisolone at a dose of 0.5–1 mg/kg may be initiated. For grade 4 cases, hospital admission is necessary, as the cutaneous presentation and secondary systemic upset are severe and potentially life-threatening. Intravenous methylprednisolone at a dose of 1–2 mg/kg should be started in this critical situation. When steroid therapy is initiated and improvement is observed, it should generally be tapered slowly over a period of more than 4 weeks. For grade 3 and above cases and those requiring hospital admission, early consultation with a dermatologist is essential to consider second-line agents in cases of steroid-refractory toxicity. Some examples of these agents include cyclosporin, infliximab and tocilizumab. In severe or refractory cases, the oncology multi-disciplinary team (MDT) may consider permanent discontinuation of the ICI treatment [28].

The incidence of neurological irAEs ranges from approximately 1% to 5%. These neurological irAEs can manifest at various times, with onset varying between 6 to 13 weeks after starting ICI therapy. There is a diverse spectrum of neurological irAEs that can affect both the central nervous system (CNS) and the peripheral nervous system [29,30]. 

Involvement of the CNS may result in conditions such as encephalitis and aseptic meningitis, whereas the peripheral nervous system can be affected by acute immune demyelinating polyneuropathy, chronic immune demyelinating polyneuropathy, cranial nerve neuropathies, myasthenic syndromes and myositis. Among neurological irAEs, neuromuscular disorders account for approximately 50% of cases, primarily including myositis, myasthenia gravis, demyelinating polyradiculoneuropathy and overlapping syndromes [29,30].

For grade 1 neurological symptoms, ICI treatment can generally be continued, and the patient should be closely monitored for any signs of deterioration. In cases of grade 2 neurological symptoms, ICI treatment should be interrupted, and the patient should be initiated on oral or intravenous corticosteroids (methylprednisolone).

For grade 3 or 4 neurological symptoms, more intensive immune modulation may be necessary, in addition to corticosteroid therapy. In some situations, corticosteroids may be exchanged for intravenous immunoglobulin (IVIG), plasma exchange, or selective separation, particularly in conditions like Guillain–Barré syndrome, leukoencephalopathy, myasthenia gravis, or immune-related myopathy [28].

Rheumatic and musculoskeletal irAEs affect approximately 10% of patients receiving ICI therapy for cancer. Among these irAEs, arthralgia and myalgia are the most common manifestations, with incidence rates ranging from 1% to 43% for arthralgia and from 2% to 20% for myalgia [31]. Arthritis is characterized by joint stiffness and swelling and can present as monoarthritis, oligoarthritis, or polyarthritis, often accompanied by tenosynovitis [32].

For grade 1, prednisone at a dose of 10–20 mg/day should be initiated and then gradually tapered upon improvement. 

For grade 2 symptoms, withdraw the treatment and refer promptly to a rheumatologist. Corticosteroid treatment can be considered (at a dose of 0.5 mg/kg). 

For grades ≥ 3, prednisone at a dose of 0.5–1 mg/kg should be initiated. In the presence of life-threatening manifestations, high-dose corticosteroids, intravenous immunoglobulin (IVIG), and/or plasma exchange/selective separation may be considered. In such cases, discontinuation of ICI therapy is always necessary [28].

However, it is important to note that each case is unique, and treatment outcomes can vary among individuals.

Based on the provided details, it appears that the irAEs of PPK and Reiter Syndrome during pembrolizumab treatment have not been reported in previous studies or in the literature. The occurrence of symptoms and their subsequent rapid improvement following corticosteroid and/or IV-IG treatment suggest an underlying neurologic and immunologic mechanism, rather than a coincidental simultaneous event. While the exact immune-mediated mechanism remains unclear, it is likely that peripheral T-cell dysregulation plays a role in these adverse events associated with pembrolizumab therapy [17]. In the management of irAEs due to ICIs like pembrolizumab, the standard approach involves discontinuing the administration of ICIs and initiating high-dose corticosteroid therapy. This has shown benefits in the majority of cases, particularly when the AEs are detected as early as possible. The addition of corticosteroid-sparing agents, such as IV-IG, may be considered on an individual basis, particularly in severe cases [2,13]. 

Close monitoring of irAEs is of the utmost importance for oncology patients undergoing ICI treatment regimens. We highly recommend a vigilant approach for monitoring irAEs in patients receiving ICI therapies. Additionally, early administration of high-dose corticosteroids is strongly advised as a treatment strategy. In cases of severe irAEs, the use of IV-IG may be considered.

Further research is essential to validate the observed trends and delve into the underlying reasons for their occurrence in patients undergoing ICI therapy. Particularly noteworthy is the identification of uncommon but potentially fatal presentations of irAEs, a finding which necessitates in-depth investigation. Understanding the timing, histopathological findings, and symptoms associated with these conditions arising due to ICI treatment is crucial, as their mechanisms remain poorly understood. Given the limited number of case reports available for patients experiencing palmoplantar keratoderma, Reiter’s syndrome, autonomic neuropathy and myasthenia gravis as irAEs, it becomes imperative to expand research efforts in this area. As the utilization of these antibodies becomes more widespread, it is of paramount importance to conduct thorough investigations into the likelihood of immune-related adverse events with these specific drugs. By conducting further studies, researchers can shed light on the mechanisms underlying irAEs and the factors contributing to their occurrence, thereby enhancing patient safety and optimizing treatment strategies for individuals undergoing ICI therapy. This concerted effort will ultimately aid in improving patient outcomes and providing better care in the context of immunotherapy.

## 5. Conclusions

Immune checkpoint inhibitors can induce various immune-related side effects. Although these occurrences are uncommon, their associated mortality rate is high. It is crucial to remain vigilant about recognizing both the typical and atypical symptoms, as well as the corresponding laboratory findings, when caring for patients. High doses of steroids typically represent the most effective treatment approach for managing any immune-related adverse events (iRAEs), and in severe cases, the consideration of intravenous immunoglobulin (IVIG) therapy should be given.

## Figures and Tables

**Table 1:** Immune checkpoint inhibitors, their indications, and side effects.

Drug	ICI Treatment	First Approved	Cancers Approved for Treatment	Most Common Side Effects
Ipilimumab	anti-CTLA-4	2011	Melanoma, RCC, CRC, HCC and NSCLC	Fatigue, diarrhea, pruritis, rash and colitis.
Pembrolizumab	anti-PD-1	2014	Melanoma, lung cancer, SCC, lymphomas, urothelial carcinoma, cancers high in MSI, MMR-deficient cancers, gastric cancers, esophageal cancers, cervical cancers, HCC, Merkel cell cancer, RCC, endometrial carcinoma, tumor mutational burden-high cancer and triple-negative breast cancer	Fatigue, musculoskeletal pain, decreased appetite, diarrhea, rash, fever, cough, constipation, nausea, abdominal pain and pruritis.
Nivolumab	anti-PD-1	2014	Melanoma, NSCLC, malignant pleural mesothelioma, RCC, classic Hodgkin lymphoma, HNSCC, urothelial carcinoma, CRC, HCC and esophageal squamous cell carcinoma	Fatigue, rash, pruritis and diarrhea.
Cemiplimab	anti-PD-1	2018	Basal cell carcinoma, NSCLC and cutaneous SCC	Nephritis, pneumonitis, hepatitis, rash, hypothyroidism or hyperthyroidism, pruritus and colitis.
Atezolizumab	anti-PDL-1	2016	Urothelial carcinoma, NSCLC, triple-negative breast cancer, SCLC, HCC and melanoma	Fatigue, nausea, vomiting, cough, dyspnea, decreased appetite, alopecia, constipation or diarrhea, headache and rash.
Durvalumab	anti-PDL-1	2017	Urothelial carcinoma and NSCLC	Fatigue, constipation, UTIs, edema, pneumonitis, dyspnea, rash, cough and nausea.

Abbreviations: RCC, renal cell carcinoma; CRC, colorectal cancer; HCC, hepatocellular carcinoma; NSCLC, non-small-cell lung cancer; SCC, squamous cell carcinoma; MSI, microsatellite instability; MMR, mismatch repair; HNSCC, head and neck squamous cell carcinoma; SCLC, small-cell lung cancer; UTIs, urinary tract infections; ICI, immune checkpoint inhibitors; PD-1, programmed cell-death 1; PDL-1, programmed death-ligand 1.

## Data Availability

Data are contained within the article or are available from the authors upon reasonable request.
